# *OsRDR6* plays role in host defense against double-stranded RNA virus, *Rice Dwarf Phytoreovirus*

**DOI:** 10.1038/srep11324

**Published:** 2015-07-13

**Authors:** Wei Hong, Dan Qian, Runhong Sun, Lin Jiang, Yu Wang, Chunhong Wei, Zhongkai Zhang, Yi Li

**Affiliations:** 1 State Key Laboratory of Protein and Plant Gene Research, College of Life Sciences, Peking University, Beijing 100871, China; 2 Institute of Plant Protection, Henan Academy of Agricultural Sciences, Henan Key Laboratory of Crop Pest Control, Zhengzhou 450002, China; 3 Ministry of Agriculture Key Lab of Southwestern Crop Gene Resources and Germplasm Innovation, Yunnan Provincial Key Lab of Agricultural Biotechnology, Biotechnology and Germplasm Resources Institute, Yunnan Academy of Agricultural Sciences, Kunming 650223, China

## Abstract

RNAi is a major antiviral defense response in plant and animal model systems. RNA-dependent RNA polymerase 6 (RDR6) is an essential component of RNAi, which plays an important role in the resistance against viruses in the model plants. We found previously that rice *RDR6* (*OsRDR6*) functioned in the defense against *Rice stripe virus* (RSV), and *Rice Dwarf Phytoreovirus* (RDV) infection resulted in down-regulation of expression of *RDR6.* Here we report our new findings on the function of *OsRDR6* against RDV. Our result showed that down-regulation of *OsRDR6* through the antisense (OsRDR6AS) strategy increased rice susceptibility to RDV infection while over-expression of *OsRDR6* had no effect on RDV infection. The accumulation of RDV vsiRNAs was reduced in the OsRDR6AS plants. In the *OsRDR6* over-expressed plants, the levels of *OsRDR6* RNA transcript and protein were much higher than that in the control plants. Interestingly, the accumulation level of OsRDR6 protein became undetectable after RDV infection. This finding indicated that the translation and/or stability of OsRDR6 protein were negatively impacted upon RDV infection. This new finding provides a new light on the function of *RDR6* in plant defense response and the cross-talking between factors encoded by host plant and double-stranded RNA viruses.

RNA silencing functions as a potent antiviral pathway in plant and animal systems^1^,^2^,^3^,^4^,^5^,^6^, and is known to be triggered by the accumulation of double-stranded viral RNA (dsRNA). The viral dsRNAs are then recognized and processed into small interfering RNAs (vsiRNAs) by distinct Dicer-like (DCL) proteins. The 21- and 22-nt vsiRNAs are known to be processed by DCL4 or its surrogate DCL2^7^,^8^. The 24-nt vsiRNAs are mainly produced by DCL3 during DNA virus (gemini- and pararetroviruses) infection in plant^7^,^9^. One strand of the vsiRNA duplex is recruited by specific Argonaute (AGO) proteins within the RNA-induced silencing complexes (RISCs) and directs the complexes for further viral RNA silencing^10^,^11^.

During the process of antiviral RNA silencing, the host RNA-dependent RNA polymerases (RDRs) contribute to secondary vsiRNAs generation^12^. The model plant *A. thaliana* possesses six RDRs^13^,^14^. RDR1 plays an important role in production and amplification of both exogenous vsiRNAs and endogenous viral activated siRNA (vasiRNA) in plants infected with positive-stranded viruses^15^,^16^,^17^. Additionally, RDR1 is involved in plant responses to abiotic stresses^18^. RDR2 has been found involved in RNA–directed DNA methylation (RdDM) pathway^19^,^20^ and required for the development of the female gametophyte^21^. RDR6 is an important component for the biogenesis of different siRNAs including vsiRNAs^22^, trans-acting siRNAs (ta-siRNAs)^23^,^24^, natural antisense-transcript-derived siRNAs (nat-siRNAs)^25^, transgene-derived siRNAs^26^, and several phased or non-phased siRNAs^27^.

Recent studies on the dicotyledonous model plant *A. thaliana* and *N. benthamiana* have demonstrated the significant role of *RDR6* in host defense response against some positive-sense single-stranded RNA viruses^28^,^29^, as well as viroid^30^,^31^. The *rdr6* mutant *Arabidopsis* plants exhibit enhanced susceptibility to the *Cucumber mosaic virus* (CMV) but not to *Turnip mosaic virus* (TuMV) or *Turnip vein clearing virus* (TVCV) infection^28^. RDR6i *N. benthamiana* plants are more sensitive to *Potato virus X* (PVX), *Potato virus Y* (PVY), CMV with Y satellite^29^ and *Potato spindle tuber viroid* (PSTVd)^31^. Tobacco plants with reduced RDR6 expression exhibit hypersusceptibility to *Turnip crinkle virus* (TCV) and *Tobacco mosaic virus* (TMV) in a temperature-dependent manner^32^. Reduced expression of NbRDR6 also permitted efficient multiplication of TMV^32^ or PVX^29^ in the shoot apices. Grafting assays indicates the requirement of NbRDR6 for symptom production induced by *Hop stunt viroid* (HSVd)^30^. We also reported previously that down-regulation of rice *RDR6* expression in rice plant increased disease symptoms caused by RSV infection comparable to that shown in the wild-type plants infected with the same virus^33^. Plant reoviruses are major threats to monocotyledonous (monocots) food crops including rice and corn. Therefore, development of new and effective disease management strategies for these viruses is critical for rice and other cereal crop production. In this study, we investigated the function of *OsRDR6* for its role in resistance against RDV, a member of genus *Phytoreovirus*.

RDV is a member of the genus *Phytoreovirus*, family *Reoviridae*. It is transmitted by leafhopper in a circulative manner and causes severe dwarf disease symptoms in rice^34^. The genome of RDV comprises twelve double-stranded RNA segments encoding seven structural proteins and at least seven nonstructural proteins^35^,^36^,^37^,^38^,^39^. To date, only a few RDV genome segments have been studied. The nonstructural Pns6 and Pns10 proteins are known as the viral cell-to-cell movement protein^39^ and viral suppressor of RNA-silencing (VSR)^40^,^41^ respectively. The outer capsid protein P2 is a multifunctional protein required for virion entry into insect vector cells^42^ and is involved in plant hormone (e.g., GA) pathway^34^,^43^. Rice encodes five RDRs: OsRDR1, OsRDR2, OsRDR3a, OsRDR3b and OsRDR6^44^. OsRDR1 is required for the production of small RNA in response to DNA-damage in rice^45^ and for the RNA silencing mediated by *Brome mosaic bromovirus* (BMV), but not by *Wheat dwarf geminivirus* (WDV)^46^. Recent report showed that OsRDR2 did not function in siR441 and siR446 production, which were previously annotated as microRNAs (miRNAs)^47^. The functions of OsRDR3a and OsRDR3b have not been studied in detail.

Although *OsRDR6* is known to play roles in the defense response against dsRNA virus infection in monocot plants, how dsRNA viruses counteract this host defense strategy remains largely unknown. We demonstrated here for the first time that down-regulation of *OsRDR6* expression in rice significantly enhanced rice susceptibility to RDV infection but up-expression of *OsRDR6* in rice had no effect on its defense against the virus. We also demonstrated that the accumulation of OsRDR6 protein in the *OsRDR6* over-expressed lines was suppressed upon RDV infection due to an unidentified mechanism that confers the suppression of translation of the transgene and/or destabilization of the protein. Taken together, our finding presented in this paper provides some new insights into the function of *OsRDR6* in defense response against dsRNA virus infection, and the defense and counter-defense reaction between host plant and virus.

## Results

### Down-regulation of *OsRDR6* expression increased rice susceptibility to RDV infection

**Figure 1:**
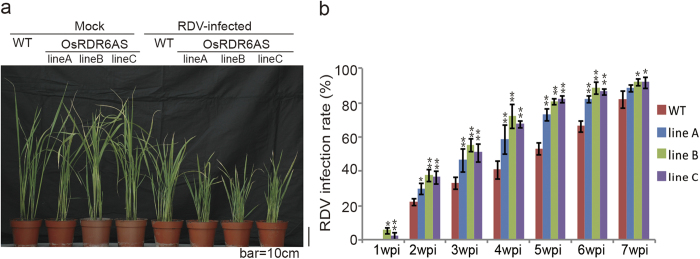
RDV-infected OsRDR6AS rice plants exhibited more severe stunting than the RDV-infected wild type (WT) Zhonghua11 plants. (**a**) Phenotypes of the RDV-infected OsRDR6AS and WT Zhonghua11 plants at 3 weeks post RDV inoculation (wpi). (**b**) Time course study of infection rate for RDV-inoculated OsRDR6AS and WT plants. The visual disease symptoms were assessed with 30 individual plants per treatment. * P < 0.05; ** P < 0.01 (Student’s t test). The inoculation assay was repeated three times. The error bars indicate the standard errors.

Our previous study showed that RDV infection in rice reduced *OsRDR6* expression^41^. We also reported in a separate study that OsRDR6AS transgenic rice plants accumulated significantly less amount of *OsRDR6* RNA transcripts and were more susceptible to RSV (a single-stranded RNA virus) infection^33^. In this study, the OsRDR6AS transgenic rice lines were inoculated with RDV via viruliferous leafhopper. By three weeks post virus inoculation (wpi), the RDV-inoculated OsRDR6AS plants exhibited more severe stunting phenotypes (Fig. 1a) than those shown by the RDV-inoculated wild type (WT) rice plants. The infection rates of RDV in the OsRDR6AS transgenic lines were also higher than those in the WT rice plants at various wpi (Fig. 1b). Plants of OsRDR6AS transgenic line B and C showed RDV symptoms as early as 1wpi (Fig. 1b). By 7wpi, the RDV infection rate in the three OsRDR6AS transgenic lines reached above 90% while the infection rate of the WT plants was about 80%, indicating that down-regulation of *OsRDR6* expression in rice increased the susceptibility to RDV infection.

### Accumulation levels of RDV RNAs correlated with RDV symptoms in the inoculated plants

**Figure 2:**
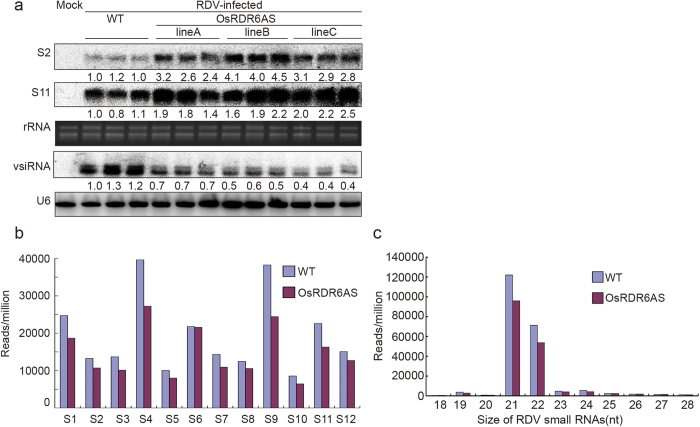
Accumulation of RDV S2 and S11 genomic RNAs and vsiRNAs in the OsRDR6AS and WT plants. (**a**) Northern blot assay of RDV S2 and S11 genomic RNAs and vsiRNAs in the OsRDR6AS and WT plants. Plants showing disease symptoms were harvested at 3 wpi. Three pools of infected plants (>10 plants per pool) were harvested from each treatment and used for total RNA isolation. The rRNA or U6 was used as loading control for the assay. (**b**) Deep sequencing result showing the relative abundance of vsiRNAs from each RDV RNA. The blue bars represent the results from the WT plants and red bars represent the results from the OsRDR6AS plants. (**c**) Size distribution of RDV-derived small RNA populations based on the deep sequencing data.

To determine whether RDV symptoms in the inoculated plants were correlated with the levels of RDV RNA accumulation in the infected plants, we analyzed RDV S2 and S11 genomic RNA levels in the assayed plants by Northern blot. Result of the assay showed that the accumulation levels of RDV S2 and S11 RNAs in the inoculated OsRDR6AS transgenic lines were much higher than that in the inoculated control WT plants (Fig. 2a). Further analysis of viral vsiRNAs accumulated in these plants revealed that the levels of RDV vsiRNAs in the inoculated OsRDR6AS transgenic lines were significantly lower than that in the inoculated control plants. This finding indicated that down-regulation of *OsRDR6* expression in rice led to a reduction of RDV RNA silencing in the RDV-infected OsRDR6AS transgenic plants.

We then analyzed the vsiRNAs from the RDV-infected OsRDR6AS and control WT plants by deep sequencing followed by data analysis using the bioinformatics tool BOAT provided by CBI (the Center of Bioinformatics) as described previously^33^,^48^. Result from two independent RDV vsiRNA libraries showed a reduced amount of RDV vsiRNAs in the inoculated OsRDR6AS plants comparable to the control plants except S6 (Table 1, Fig. 2b, Fig. S1). The length and polarity profiles of vsiRNAs from the infected OsRDR6AS plants were similar to that from the wild type plants (Fig 2c). Unlike the previous report for RSV^33^, the RDV derived vsiRNAs were mostly 21- and 22-nt in length (Fig. 2c). This result suggested that *OsRDR6* acted differently during silencing double-stranded RNA viruses and negative-sense single-stranded RNA viruses.

### Over-expression of *OsRDR6* in rice did not affect RDV infection

**Figure 3:**
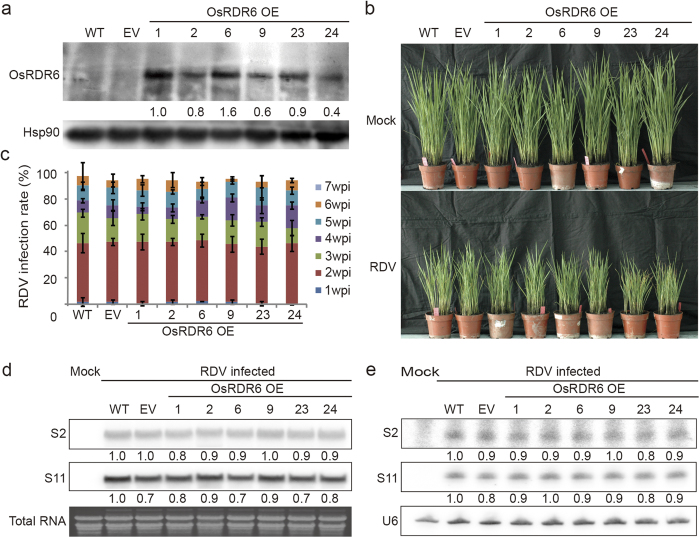
Over-expression of *OsRDR6* did not affect RDV infection in rice. (**a**) Western blot analysis of OsRDR6 protein expression in the *OsRDR6*-over-expression (OsRDR6 OE), empty vector transformed (EV) and the WT rice plants. Rice Hsp90 was served as the loading control. (**b**) Comparison of RDV infected phenotypes in the OsRDR6 OE, EV and WT plants. The OsRDR6 OE rice plants did not show reduced disease symptoms comparable with the control plants. (**c**) The infection rates of the OsRDR6 OE, EV and WT plants at various wpi. The infection rate was determined using 30 individual plants per treatment at each wpi. Inoculation assay was repeated three times. The error bars indicate the standard errors. P > 0.05 (Student’s t test). (**d**) Northern blot assay of RDV S2 and S11 genomic RNA accumulation in the OsRDR6 OE, EV and WT plants. Thirty plants showing disease symptoms were harvested from each treatment at 3 wpi for total RNA isolation. (**e**) Northern blot assay for RDV siRNAs accumulation in the OsRDR6 OE, EV and WT plants. The plant samples used in (**d**) were used for this analysis. U6 RNA was used as the loading control.

Because silencing *OsRDR6* expression in rice resulted in an increased RDV accumulation and stronger disease symptoms in the RDV-infected rice plants, we reasoned that over-expression of *OsRDR6* would cause an increased resistance against RDV. To test this hypothesis we generated *OsRDR6-*over-expressing transgenic rice lines and inoculated them with RDV. Figure S2 showed that transgenic line 1, 2, 6, 23 and 24 contained a single-copy of *OsRDR6* transgene while line 9 has two copies (Fig. S2). Western blot showed that all the over-expression (OE) transgenic lines produced high levels of OsRDR6 proteins while this protein in the WT or the empty vector (EV) transformed control rice plants was not detectable with this method (Fig. 3a). Growth of all the OsRDR6 OE lines were comparable to the WT and EV control plants (Fig. 3b top panel). Surprisingly, none of the OsRDR6 OE rice lines showed an increased resistance to RDV infection comparable with the WT and EV control plants (Fig. 3b). In addition, the infection rates of all the RDV-inoculated OsRDR6 OE lines were similar to that of the WT and EV control plants (Fig. 3c).

To further compare the OsRDR6 OE lines with the control plants, we examined RDV S2 and S11 RNA accumulation in these plants through Northern blot assay. Result of the assay showed that the accumulations of S2 and S11 RNA in the RDV-inoculated OsRDR6 OE lines were similar to those in the WT and EV control plants (Fig. 3d). Northern blot analysis of vsiRNAs also showed that the accumulation levels of vsiRNAs in the OsRDR6 OE lines were similar to those in the RDV-inoculated WT and EV control plants (Fig. 3e). This finding indicated that increased OsRDR6 protein accumulation in OE rice plants did not improve its resistance to RDV infection.

### RDV infection reduced *OsRDR6* expression in the OsRDR6 OE lines

**Figure 4:**
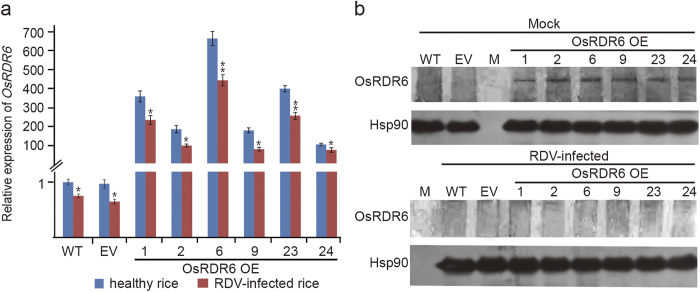
RDV infection reduced the expression of *OsRDR6* in the *OsRDR6*-over-expression rice plants. (**a**) Relative expression levels of *OsRDR6* RNA transcript in the OsRDR6 OE, EV and WT plants. Relative transcript levels were calculated using the 2^−ΔΔC(t)^ method and *OsEF*-1a gene transcripts as the internal control. The error bars indicate the standard errors. Asterisks indicate P values compared with healthy rice plants: * P < 0.05; ** P < 0.01 (Student’s t test). (**b**) Western blot assay of OsRDR6 proteins accumulation in OsRDR6 OE, EV and WT plants. Expression of rice Hsp90 protein was used as the loading control.

To investigate the effect of RDV infection on *OsRDR6* expression in rice plants, we analyzed *OsRDR6* mRNA and protein accumulation in the RDV-inoculated and non-inoculated OsRDR6 OE, EV and WT rice plants through quantitative RT-PCR. Results of the assay showed that the levels of *OsRDR6* mRNA were much higher in the OE plants than those in the WT or EV control plants (Fig. 4a). Interestingly, RDV infection resulted in decreased levels of *OsRDR6* transcripts in all assayed plants. Western blot assay agreed with the quantitative RT-PCR result and showed that the accumulation of OsRDR6 protein in the OsRDR6 OE lines was clearly decreased after RDV infection (Fig. 4b). These data indicated that over-expression of *OsRDR6* in rice plant had no significant effect on host response to RDV infection, and the decreased accumulation of OsRDR6 protein in the RDV-infected OE lines might be caused by a translational suppression of OsRDR6 and/or the stability of the protein.

## Discussion

Many studies have shown that the *RDR6-*dependent RNA silencing pathway plays an important role in plant defense against virus infection. For example, *A. thaliana rdr6* mutant plants showed an increased susceptibility to CMV infection^26^,^28^. Further study by Zhu *et al.* showed that *RDR6*-dependent silencing of CMV in *N. benthamiana* was initiated by CMV Satellite RNA-derived small interfering RNA known as satsiR-12^49^. In other studies, TMV derived vsiRNAs were found to be significantly reduced in the TMV-infected *A. thalianardr 6* mutants^32^,^50^. *RDR6* of *N. benthamiana* was also shown to regulate defense response against systemic spreading of TCV^32^ and invasion of apical meristem by *Potato virus X*^29^. Our previous study also demonstrated that knockdown of *OsRDR6* expression in rice increased its susceptibility to RSV infection^33^. In addition, *RDR6* was reported to play an important role in disease resistance against viroids. For example, silencing *RDR6* gene expression in *N. benthamiana* enhanced disease symptoms caused by HSVd^30^. Also, the accumulation of PSTVd in the NbRDR6-silenced *N. benthamiana* plants was increased early after PSTVd infection than that in the non-silenced *N. benthamiana* plants^31^. A recent report by Wang *et al.* showed that over-expression of *Gossypium hirsutum L*. (cotton) *GhRDR6* in *N*. *benthamiana* enhanced *N*. *benthamiana* resistance to *Potato virus Y* infection^51^. Interestingly, results shown in this paper indicated that over-expression *OsRDR6* in rice had no clear influence on RDV infection, based on the infection rates of RDV in the OsRDR6 OE, EV and WT control rice plants (Fig. 3c). Our North blot assay supported the above symptom observation and showed that the accumulation levels of RDV S2 and S11 RNAs were similar between the OsRDR6 OE lines and the WT or EV control lines (Fig. 3d). In addition, the accumulation levels of S2 and S11 vsiRNAs in the OsRDR6 OE lines were comparable to those from the control plants (Fig. 3e). Taken together, our results provided new insight into the function of host *RDR6* in antiviral RNA silencing during virus infection. We speculate that over-expression of *OsRDR6* in rice may alter the expression of other host genes that are also involved in the RNA silencing pathway. It is also possible that dsRNA viruses encode other factors that influence *RDR6* gene function and regulate the accumulation of dsRNA viruses in its host plants.

**Figure 5:**
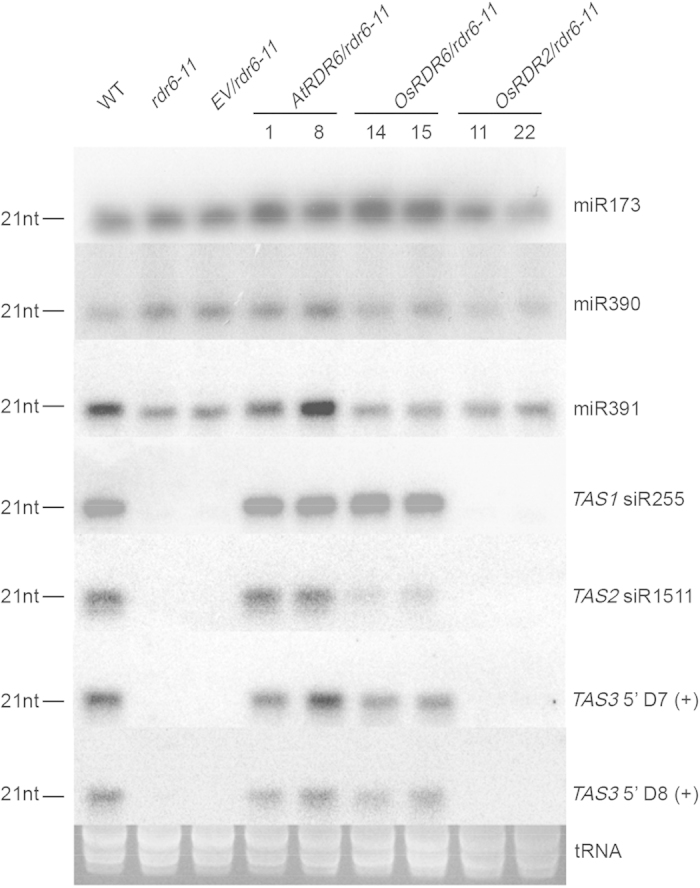
Over-expression of *OsRDR6* and *AtRDR6* in *rdr6* mutant *A. thaliana* restored the accumulation of *TAS* siRNAs in plants. Four *TASs* [*TAS1* siRNA255, *TAS2* siRNA1511, *TAS3* 5'D7 (+) and *TAS3* 5'D8 (+)] were analyzed using Northern blot assay. Accumulation of miR173, miR390 and miR391 in the *OsRDR6*/*rdr6–11*, *AtRDR6*/*rdr6–11* or *OsRDR2*/*rdr6–11* transgenic *Arabidopsis* plants were also analyzed in the assay. tRNA isolated from each sample was served as the loading control.

To verify the biological function of OsRDR6 protein in the OE lines, we conducted a complementation assay by over-expressing the full length coding sequence of *OsRDR6* and *AtRDR6* in the *rdr6*–11 mutant *Arabidopsis*^23^,^24^ to produce *OsRDR6*/*rdr6–11* and *AtRDR6*/*rdr6–11* transgenic lines, respectively. *OsRDR2*/*rdr6–11* line was produced to serve as a control. Analysis of these lines showed that *OsRDR6* and *AtRDR6* indeed restored the accumulation of *TAS* siRNAs^52^ in the over-expression *Arabidopsis* transgenic plants whereas the transgenic plants over-expression *OsRDR2* did not (Fig. 5). Therefore the inability of OsRDR6 protein in the OE rice line to suppress RDV infection was not due to the authenticity of gene sequence transformed into the rice plants.

The antiviral RNA silencing machinery also contains many other proteins involved in different process including DCLs^53^,^54^,^55^,^56^ and AGOs^56^,^57^,^58^,^59^. For instance, recently we found that AGO18 confers broad-spectrum resistance against single-stranded RNA virus RSV and double-stranded RNA virus RDV through a novel mechanism of positive regulation AGO1 homeostasis^6^. The broad-spectrum resistance of AGO18 provides a potential application of AGO18 in crops. Both transcript and translation levels of *AGO18* were increased after RDV infection. While unlike *AGO18*, the quantitative RT-PCR data shown in Fig. 4a indicated that *OsRDR6* mRNA levels were reduced after RDV infection. Although the transcript levels of *OsRDR6* in the OE rice lines infected with RDV were reduced to some extent, these expression levels were still over a hundred fold higher than those observed from the control plants. Result of Western blot assay (Fig. 4b top panel) showed that OsRDR6 protein was indeed accumulated in the OE lines but not in the WT or EV transformed control plants before RDV inoculation. However, after RDV inoculation, OsRDR6 protein in the OE lines also became undetectable (Fig. 4b bottom panel). Because *RDR6* plays an important role in antiviral RNA silencing pathway, it is also a target of virus-encoded suppressor of RNA silencing (VSRs). For example, *Rice yellow stunt rhabdovirus* (RYSV) encoded P6 protein was reported to interact with OsRDR6 and AtRDR6, and to repress the synthesis of the secondary siRNAs in both *N. benthamiana* and rice protoplasts^60^. Li *et al.* reported that expression of βC1 by the DNA satellite associated with *Tomato yellow leaf curl China virus* (TYLCCNV, geminivirus) induced the expression of *Nbrgs-CaM* leading to a repression of *RDR6* expression in *N. benthamiana* followed by suppressing the TYLCCNV-induced gene silencing^61^. We also reported that RDV encoded Pns10 suppressed the expression of *NbRDR6* in *N. benthamiana* plant^41^. Interestingly, in this study, infection of RDV in the *OsRDR6*-over-expression rice plants significantly reduced the accumulation of OsRDR6 protein. We hypothesized that the RDV encoded Pns10 might play a role in suppression of translation of *OsRDR6*. To test this hypothesis we performed a binding assay using purified recombinant Pns10 protein and *RDR6* mRNA. The results of the assay showed that Pns10 protein did not bind *RDR6* mRNA. In addition, Pns10 protein did not interact with OsRDR6 protein in the yeast two-hybrid and co-immunoprecipitation (CO-IP) assays. Whether other uncharacterized RDV encoded proteins contain such a function of suppress translation of OsRDR6 and/or destabilize the protein remains to be determined. To test whether other virus infection of rice also affects Os*RDR6* expression, we analyzed the mRNA and protein level of *OsRDR6* in RSV infected rice and found that infection of RSV had no effect on *OsRDR6* expression. Therefore, we presume that viruses with different genome structures may have different effects on *OsRDR6* expression.

Although over-expression of *OsRDR6* in rice failed to alter plant resistance to RDV infection, down-regulation of *OsRDR6* expression indeed enhanced plant susceptibility to the virus comparable with the WT control plants. Our result agreed with previous reports that *OsRDR6* did play an important role in plant defense against dsRNA viruses. Further characterization of dsRNA virus encoded proteins will help us to better understand the mechanism(s) that regulate the expression of *OsRDR6* and stability of the protein in the OE transgenic lines upon RDV infection.

## Methods

### Rice transformation and RDV infection assay

OsRDR6AS transgenic rice lines A to C were from a previously described source^33^. To generate *OsRDR6*-over-expression rice, the full-length coding sequence of *OsRDR6* was ligated into the *Pst*I site within the binary vector pCam23ACT:OCS^62^ to produce pCam23-ACT:OCS-OsRDR6. Briefly, the full-length coding sequence of *OsRDR6* was digested with *Hind*III (Promega) from pBS(KS)-OsRDR6^33^. Then the ends made blunt with T4 DNA polymerase (Promega) and digested with *Xba*I (Promega). pCam23ACT:OCS was digested with *Pst*I (Promega). Then the ends made blunt with T4 DNA polymerase (Promega) and digested with *Pst*I (Promega). The above digested *OsRDR6* sequence and pCam23ACT:OCS were ligated by T4 DNA Ligase (NEB). The plasmid was transformed into a widely cultivated rice cultivar (Zhonghua11) in China by Weiming Kaituo Co., Ltd (Beijing, China) through *A. tumefaciens*-mediated stable transformation procedure. Seeds of T0 generation rice were immersed in water containing G418 antibiotic (10 mg/L) to obtain positive transgenic plants. The *OsRDR6* transgenic and empty vector transformed control rice plants were grown in a greenhouse under a 14-h light/10-h dark photoperiod at 24–29 °C. Thirty T1 generation transgenic rice plants from each line were inoculated with RDV using viruliferous insect (*Nephotettix cincticeps*) as previously described^43^,^48^.

### *OsRDR6* and *AtRDR6* functional complementation assay

*OsRDR6*, *OsRDR2* and *AtRDR6* were cloned individually into pWM101 to produce pWM101-*OsRDR6*, *pWM101-OsRDR2* and *pWM101-AtRDR6*. The resulting plasmids were transformed into *A. thaliana rdr6–11* mutant plants using the floral-dip method^63^. Seeds from the transgenic *OsRDR6*/*rdr6–11*, *OsRDR2*/*rdr6–11* and *AtRDR6*/*rdr6–11 A. thaliana* plants were sterilized and then planted on a 1/2 MS medium containing hygromycin (25 mg/L). The germinated seedlings were transferred to a greenhouse at 10 days post planting. Individual transgenic lines were determined through RT-PCR with specific primers (Table S1). Total RNA was then extracted from more than thirty plants with different transgenes and used for small RNA assay through Northern blot with specific probes (Table S2)

### OsRDR6 protein expression, polyclonal antibody production and Western blot assay

A 1221-bp sequence in the 3' end of *OsRDR6* open reading sequence was cloned into the expression vector pET-28a(+) prior to transformation into *Escherichia coliBL21*(*DE3*) using primers OsRDR6F3-3/OsRDR6R3-3 (Table S1). The transformed cells were cultured at 37 °C and then induced for 4h with 0.5 mM isopropyl-β-d-1-thiogalactoside (IPTG). Cultures of *E.Coli* were harvested and sonicated at 4 °C. Inclusion bodies were isolated through HiTrap Chelating with the FPLC System as instructed by the manufacture (Amersham). The extracted recombinant proteins were separated in 8% SDS-PAGE gel. Polyclonal antiserium against OsRDR6 was raised in rabbit by ComWin Biotech Co., Ltd (Beijing, China). Plant protein extracts were separated in 8% SDS-PAGE gels and transferred to polyvinylidene fluoride membranes as instructed (Immobilon-P Transfer membrane, MILIIPOR). The membranes were blocked and probed with the anti-OsRDR6 antiserium diluted 1:1,000 in blocking buffer (1×TBS, 0.1% Tween-20, 5%BSA). Anti-Rice Hsp90 was purchased from HuaDa (Beijing, China) diluted 1:2,000 in blocking buffer and used as protein loading controls.

### Southern blot analysis

Southern blot assay was similar as previously described^64^. Briefly, forty microgram total genomic DNA was obtained from each transgenic line, digested overnight with *Hind*III restriction enzyme and transferred to Hybond-N+ nylon membrane (Amersham) through electrophoresis. Probes specific for the hygromycin-resistance gene (Hyg) were amplified with the Hyg-specific primers Hyg-F/R (Table S1), and labeled using the DIG DNA labeling mix kit as instructed (Roche). Hybridization was carried out overnight at 42 °C in an ULTRAhyb^®^ hybridization buffer (Ambion) and then detected with an anti-DIG antibody conjugated with alkaline phosphatase (Roche).

### Non-preference test

Non-preference tests were performed using rice seedlings with different genetic background. The seedlings were inoculated with the virus viaviruliferous leafhopper vector as described previously^65^.

### RT–PCR

Total RNA was extracted from *A. thaliana* plants using Trizol as instructed (Invitrogen) followed by the DNase treatment using RQ1 RNase-free DNase (Promega). Synthesize of cDNA was conducted using the Super Script III Reverse Transcriptase (Invitrogen) and an oligo d (T) primers instructed by the manufacture. The resulting cDNA was diluted 20 fold with water and subjected to 30 cycles of PCR reaction with gene-specific primers (Table S1). Expression level of *AtelF4A* was used as an internal control.

### Quantitative real-time PCR

The rice plants showing typical disease symptoms were collected at 3 wpi. Total RNA was extracted from more than ten rice plants as a pool. The RNA for the quantitative RT-PCR was treated with RQ1 RNase-free DNase (Promega) for 30 min at 37 °C followed by phenol/chloroform extraction. Reverse transcription was done using two microgram RNA per twenty microliter reaction with the Super Script III Reverse Transcriptase and an oligo d(T) primer. The quantitative PCR was performed using the SYBR Green Real-Time PCR Master Mix as instructed (TOYOBO) and primers specific for *OsRDR6* (OsRDR6 P13/P14, Table S1). Expression of OsEF-1a gene was used as an internal control for the assay (using primers OsEF1a F/R, Table S1). Three independent biological replicates were analyzed per treatment. Relative transcription levels were calculated using 2^-ΔΔC(t)^ method.

### Determination of viral S2 and S11 genomic RNA accumulation

Accumulation of RDV S2 and S11 genomic RNA was analyzed by Northern blot as described^33^. Briefly, ten microgram total RNA from each assayed plant was transferred to a Hybond N^+^ nylon membrane (Amersham) after electrophoresis in a 1.2% (w/v) formaldehyde-denaturing agarose gel. Radioactive probes used for the detection were PCR products amplified with primer sets S2-F1/S2-R1 and S11-F1/S11-R1 (Table S1) and labeled with α−^32^P-dCTP using the Random Primer DNA Labeling Kit Ver.2 (Takala). The hybridization was performed overnight in the PerfectHyb^TM^ Plus Hybridization Buffer (Sigma) at 65 °C. The blotted membrane was washed for 15 min in a 2 ×SSC solution containing 0.1% SDS at 65 °C followed by washing two times (15 min each) in a 0.1×SSC solution containing 0.1% SDS. The detection signal was monitored with a phosphorimager (GE, USA) and quantified using the ImageJ (version 1.44).

### Detection of Small RNAs

The rice plants of 30 seedlings showing typical disease symptoms were pooled at 3 wpi. Small RNAs were extracted using RNAzol®RT as instructed (Molecular Research Center, Inc. USA). Ten microgram small RNA from each sample was separated in a 15% denaturing polyacrylamide gel containing 8 M urea and transferred to a Hybond N+ nylon membrane (Amersham). Approximately 500 ng PCR product amplified with the S2-F1/S2-R1 or S11-F1/S11-R1 primer set (Table S1) and labeled with α−^32^P-dCTP were used as the probes. Hybridization was performed overnight in the PerfectHyb^TM^ Plus Hybridization Buffer (Sigma) at 42 °C. The membrane was stripped with 0.1% SDS at 100 °C 10 min three times to remove S11 probe and hybridized with S2 probe.

### Small RNA sequencing and bioinformatics analysis

More than fifteen seedlings were harvested from each treatment and pooled for RNA isolation. The procedure of small RNA library construction and Illumina1G sequencing was described previously^33^,^48^. Sequences of 18–28 nt small RNAs were mapped to the *O. sativa* genome sequence (TIGR Rice Annotation Release 5.0, ftp://ftp.plantbiology.msu.edu/pub/data/EukaryoticProjects/osativa/annotationdbs/) and RDV genome sequence (ftp://ftp.ncbi.nih.gov/genomes/Viruses/Ricestripevirusuid14795/)^66^ using the bioinformatics tool BOAT provided by CBI (the Center of Bioinformatics).

## Additional Information

**How to cite this article**: Hong, W. *et al.* *OsRDR6* plays role in host defense against double-stranded RNA virus, *Rice Dwarf Phytoreovirus*. *Sci. Rep.* **5**, 11324; doi: 10.1038/srep11324 (2015).

## Supplementary Material

Supplementary Information

## Figures and Tables

**Table 1:** Summary of the deep sequencing results for the small RNAs derived from the RDV-infected rice plants.

**Libraries**	**Replicate 1**		**Replicate 2**	
	**Zhonghua11**	**OsRDR6AS**	**Zhonghua11**	**OsRDR6AS**
Unique sequences^a^	2,519,939	2,171,452	2,527,417	1,910,802
Total reads^a^	8,504,431	7,264,974	6,756,834	6,032,770
Unique RDV vsiRNA^a^,^b^	124,143	122,007	142,329	110,547
Total Reads of RDV vsiRNA^d^ (% of total reads)^a^,^b^	1,886,645 (22.18%)	1,265,099 (17.41%)	1,584,898 (23.46%)	1,071,029 (17.76%)
(+)-strands (%)^a^,^c^	59.40%	61.00%	60.50%	62.70%
(−)-strands (%)^a^,^d^	40.50%	39.00%	39.50%	37.30%

^a^Number of sequences within the set (18 nt≤ length ≤28 nt).

^b^Sequences with perfect matches to the RDV genome.

^c^Sequences with perfect matches to the (+)-strand RNAs of the RDV genome.

^d^Sequences with perfect matches to (−)-strand RNAs of the RDV genome.
