# Supplementary material for: OsRDR6 plays role in host defense against double-stranded RNA virus, Rice Dwarf Phytoreovirus
Source: Sci Rep. 2015 Jul 13;5:11324. doi: 10.1038/srep11324 (PMC4499934; doi:10.1038/srep11324)
Supplement: Supplementary Information [file srep11324-s1.pdf]

Supplementary Information

OsRDR6 plays role in host defense against double-stranded RNA virus, *Rice Dwarf Phytoreovirus*

Wei Hong[1]‡, Dan Qian[1]‡, Runhong Sun[1,2], Lin Jiang[1], Yu Wang[1], Chunhong Wei[1], Zhongkai Zhang[3]*, Yi Li[1]*

[1]State Key Laboratory of Protein and Plant Gene Research, College of Life Sciences, Peking University, Beijing 100871, China.

[2]Institute of Plant Protection, Henan Academy of Agricultural Sciences, Henan Key L aboratory of Crop Pest Control, Zhengzhou 450002, China.

[3]Ministry of Agriculture Key Lab of Southwestern Crop Gene Resources and Germplasm Innovation, Yunnan Provincial Key Lab of Agricultural Biotechnology, Biotechnology and Germplasm Resources Institute, Yunnan Academy of Agricultural Sciences. Kunming 650223, China.

*Corresponding author: Dr. Yi Li, College of Life Sciences, Peking University, Beijing, 100871, China. Telephone number: 86-10-62759690. Fax number: 86-10-62756903. E-mail address: liyi@pku.edu.cn

Dr. Zhongkai Zhang,Ministry of Agriculture Key Lab of Southwestern Crop Gene Resources and Germplasm Innovation, Yunnan Provincial Key Lab of Agricultural Biotechnology, Biotechnology and Germplasm Resources Institute, Yunnan Academy of Agricultural Sciences, Kunming, 650223, China. Telephone number: 86-871-65183204.Fax number: 86-871-65160084. E-mail address: zhongkai99@sina.com

‡Both authors contributed equally to this work.

Figure S1. Genomic view of RDV vsiRNAs from RDV-infected rice plants. The vsiRNAs per million of the total sequenced small RNAs were plotted against the (+)-(top) or (-)-(bottom) strands of RDV genomic RNAs (S1-S12) using a single-nucleotide window.

Figure S2. Southern blot assay of *OsRDR6*-over-expression lines. Probes were amplified with the Hyg-specific primers Hyg-F/R (Table S1) which are specific to hygromycin-resistance gene, hygromycin phosphotransferase (Hyg) and labeled by DIG DNA labeling mix. Except line 9, other five transgenic lines are single copy T-DNA inserted. WT, Zhonghua 11 wild type rice; EV empty vector transformed Zhonghua 11; OsRDR6 OE,*OsRDR6*-over-expression lines; M, DNA marker.

Figure S3. RT-PCR screening of transgenic *A. thaliana* lines. (a) Detection of *OsRDR*6 transcript expression in transgenic *rdr*6 *A. thaliana* (*rdr6-11*) lines over-expressing *OsRDR6*. Lines 14 and 15 are two independent lines**. (b)** Detection of *OsRDR*2 transcript expression in transgenic *rdr*6 *A. thaliana* (*rdr6-11*) lines over-expressing *OsRDR2*. Lines 11 and 22 are two independent lines**. (c)** Detection of *AtRDR*6 transcript expression in transgenic *rdr*6 *A. thaliana* (*rdr6-11*) lines over-expressing *AtRDR6*. Lines 1 and 8 are two independent lines. WT, Columbia (Col) wild type *A. thaliana* plants. EV, *A. thaliana* plant lines transformed with pWM101 plasmid vector. +RT, the RNA sample treated with reverse transcriptase. –RT, the RNA sample that did not treated with reverse transcriptase for exclusion of the possibility of genomic DNA contamination. *AteIF4A* was used as an internal control.

Figure S1

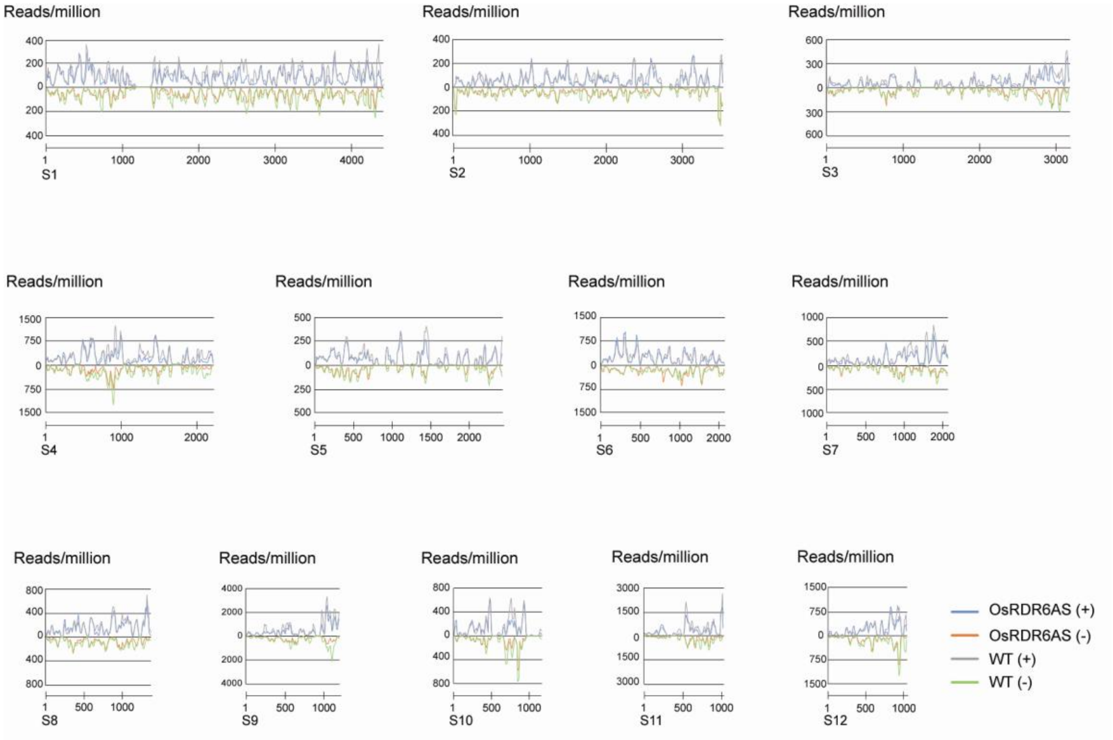
Reads/million
S1
S2
S3
S4
S5
S6
S7
S8
S9
S10
S11
S12
OsRDR6AS (+)
OsRDR6AS (-)
WT (+)
WT (-)

Figure S2

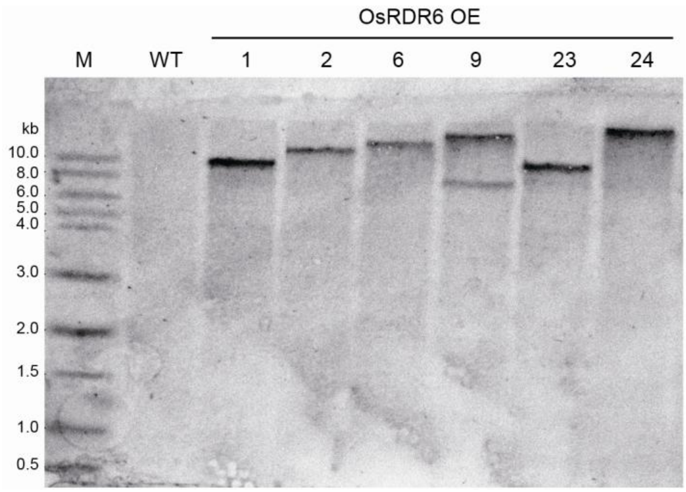

Figure S3

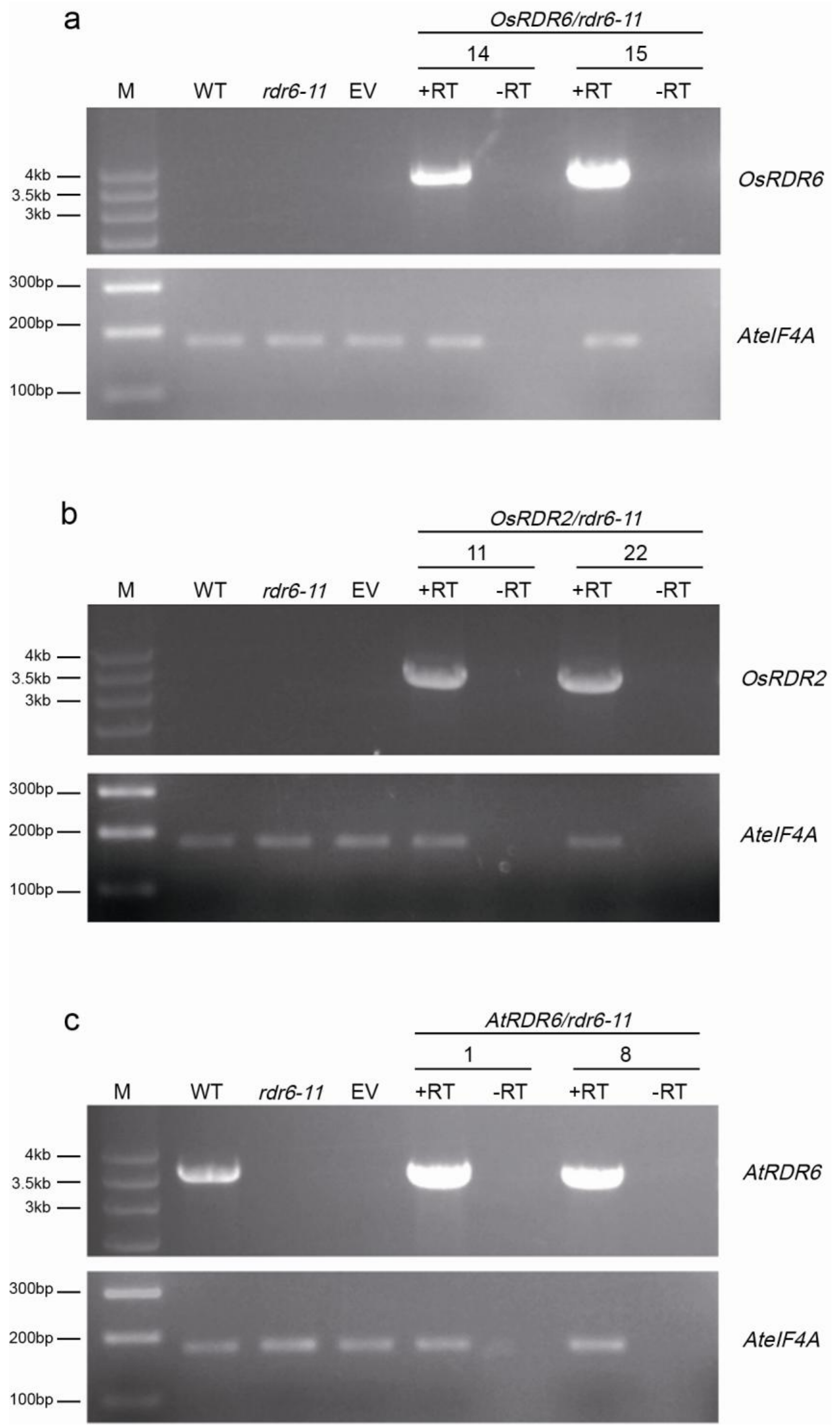
a
OsRDR6/rdr6-11
14
15
M
WT
rdr6-11
EV
+RT
-RT
+RT
-RT
4kb
3.5kb
3kb
OsRDR6
300bp
200bp
100bp
AteIF4A
b
OsRDR2/rdr6-11
11
22
OsRDR2
c
AtRDR6/rdr6-11
1
8
AtRDR6

Table S1 Specific primers used in this study

**Primers for OsRDR6 polyclonal antibody production**	
OsRDR6F3-3	5’-GAGCTCTTGAATAGTTATTTTGTTAAGCATG-3’
OsRDR6R3-3	5’-AAGCTTTCATATCCTCTCGGAGATGTATG-3’
**Primers for amplifying probes for Southern blot**	
Hyg-F	5’-CTTCTGCGGGCGATTTGT -3’
Hyg-R	5’-ATTGACTGGAGCGAGGCG-3’
**Primers for amplifying viral genomic RNA as DNA probes for Northern blot**	
S2-F1	5’-GGCAAAACCTCGCCATGGCT-3’
S2-R1	5’- AAAGCAAGCAGGGTGAATAGGTAG-3’
S11-F1	5’-TTTGAGTTCTGACAGTGATGCCAATAC-3’
S11-R1	5’-ATCATATATGAGTGGGTGTTTTTGGCTT-3’
**Primers for quantitative real-time PCR**	
OsRDR6 P13	5’-TAAGAACAGCAGCAAGAAGCAAG-3’
OsRDR6 P14	5’-ATAGACAGCCCTCAACTGAAACG-3’
OsEF1a-F	5’-ACATTGCCGTCAAGTTTGCTG-3’
OsEF1a-R	5’-AACAGCCACCGTTTGCCTC-3’
**Primers for RT-PCR screening for transgenic *A. thaliana* lines**	
AteIF4A-RT-F	5’-GCGCATCCTCCAAGCTGGTGTCC-3’
AteIF4A-RT-F	5’-GGTGGAAGAAGCTGGAATATGTCAT-3’
AtRDR6-RT-F	5’-CCGGAATTCATGGGGTCAGAGGGAAATATGAAG-3’
AtRDR6-RT-R	5’-CGGGGTACCGCGAGACGCTGAGCAAGAAACTTAGCC-3’
OsRDR6-RT-F	5’-GAATTCGATCACCCAAGCCAT-3’
OsRDR6-RT-R	5’-TCATATCCTCTCGGAGATGTATG-3’
OsRDR2-RT-F	5’-AGTCCCCAAACCTCCCATAC-3’
OsRDR2-RT-R	5’-TCAGGCGCTGCAGTCCATCTGCA-3’

Table S2 Probes for small RNA Northern blot in *OsRDR6* and *AtRDR6* functional complementation assay

miRNA173	5’-GTGATTTCTCTCTGCAAGCGAA-3’
miRNA390	5’-GGCGCTATCCCTCCTGAGCTT-3’
miRNA391	5’-TGGCGCTATCTCTCCTGCGAA-3’
TAS1 siR255	5’-TACGCTATGTTGGACTTAGAA-3’
TAS2 siR1511	5’-AAGTATCATCATTCGCTTGGA-3’
TAS3a D7 (+)	5’-GGGGTCTTACAAGGTCAAGAA-3’
TAS3a D8 (+)	5’-AAGGCCTTACAAGGTCAAGAA-3’
